# Flow Cytometry Coupled with Resuscitation Assays As a High-Resolution Tool to Inform Environmental Management and Disinfection of Settings Affected by Tuberculous Mycobacteria

**DOI:** 10.3390/microorganisms12061068

**Published:** 2024-05-25

**Authors:** André C. Pereira, Mónica V. Cunha

**Affiliations:** 1 Centre for Ecology, Evolution and Environmental Changes (cE3c), CHANGE—Global Change and Sustainability Institute, Faculdade de Ciências, Universidade de Lisboa, 1749-016 Lisboa, Portugal; andre.c.pereira94@gmail.com; 2 Biosystems & Integrative Sciences Institute (BioISI), Faculdade de Ciências, Universidade de Lisboa, 1749-016 Lisboa, Portugal

**Keywords:** mycobacteria, dormancy, flow cytometry, environmental contamination, disinfection

## Abstract

Environmental decontamination and water disinfection practices are hallmarks of disease prevention and control in agricultural and public health settings. Informed fit-to-purpose biocontainment is thus dependent on methodologies accurately assessing microbial burden and viability. Also, rigorous evaluation of the efficacy of biocontrol measures implies monitoring microbial inactivation after decontamination/disinfection procedures. In this study, we used flow cytometry coupled with a resuscitation protocol to monitor the metabolic inactivation of bacteria capable of entering non-cultivable states, after the application of a chlorine-based water disinfectant. For this purpose, we used *Mycobacterium bovis* BCG as a model of slow-growing bacteria able to enter dormancy and representing a multi-host pathogen in a zoonotic disease system—animal tuberculosis—thriving both across temperate and semi-arid regions and involving environmental contamination. The biocide activity of a commercial sodium dichloroisocyanurate (NaDCC) disinfectant against *M. bovis* BCG was evaluated through mock environmental matrix tests. Using the manufacturer-recommended dosage of NaDCC, BCG cells were apparently inactivated after 24 h upon exposure. However, we show via flow cytometry that, upon exposure to optimal growth conditions, mycobacterial cells were able to regain metabolic activity shortly after, highlighting a sublethal effect of NaDCC at the recommended commercial dosage due to reversible BCG cell damage. In contrast, increasing twice the disinfectant dosage completely inactivated BCG cells after 24 h of exposure, with full irreversible loss of metabolic activity. Methodological workflows based on conventional culture or PCR would have missed the detection of these dormant subpopulations that were in fact able to resume growth when following the recommendations of a commercial disinfectant. This study highlights the superior, high-resolution value of single-cell approaches, such as flow cytometry, to accurately assess the activity of biocides against metabolically heterogeneous and dormant pathogenic bacteria with environmental cycles, supporting data-driven prioritization of environmental management and disinfection options in contaminated vulnerable settings.

## 1. Introduction

Global warming has been aggravating existing vulnerabilities across the globe, including the natural semi-arid systems within the Iberian Peninsula, leading to land degradation and drought, and reducing the ability of species to cope with nutrient and water shortages [1,2,3]. These Iberian Peninsula ecosystems have a high degree of human intervention, with intensification of livestock management but also of wild ungulate populations for hunting purposes to increase harvest yields and profits [4,5,6]. Artificial management practices of wild ungulates include introduction/restocking, fencing, construction of waterholes for water provision, and artificial feeding and watering [7,8,9]. These measures spatially concentrate and elevate animal densities, especially during the summer, when resource shortages heighten [10]. The aggregation of ungulates increases the frequency of both direct and indirect animal contacts [11]. These are responsible for the transmission of infectious agents, namely, slow-growing mycobacteria, such as *Mycobacterium bovis*, causing animal tuberculosis [12]. The extensive husbandry of cattle with interfaces of contact with wildlife further increases the risk of mycobacteria transmission [13]. Moreover, artificial feeding and drinking stations for cattle attract wildlife to farms [14], creating windows of opportunity for interspecific transmission. Microbial contamination of pasture, food, water, and other natural substrates with droplets and fluids from animals shedding mycobacteria and other pathogens is believed to play a major role in the bidirectional transmission of *M. bovis* via the environment [15,16,17].

In livestock production and artificial wildlife management, water quality and safety need to be guaranteed [18,19,20], often by the addition of chlorine disinfectants in drinking stations or waterholes. Chlorination is mostly employed on an empirical basis, with few tools for managers to monitor the efficacy of their practices.

Most workflows detecting and quantifying waterborne pathogenic bacteria and microbial indicators of water quality rely on culture: the most probable number (MPN) technique and multiple tube fermentation tests, membrane filtration, and pour or spread plate counts [21,22]. The first technique is based on the incubation of serially diluted samples in broth media in multiple tubes. Positive results are assigned to tubes exhibiting bacterial growth and, eventually, gas formation, followed by a comparison with the MPN table and calculation of microorganism concentration [21,22]. The second technique is based on the incubation on solid media of a membrane previously used to filtrate the liquid sample, with counting of bacterial colonies and calculation of the number of colony-forming units (CFUs) [21,22]. The third technique is based on sample homogenization and incubation on a solid medium followed by calculating the number of CFUs [21,22].

Additionally, molecular-based methods, such as polymerase chain reaction (PCR), enzyme-linked immunosorbent assay (ELISA), or enzymatic-based tests, such as adenosine triphosphate (ATP) analysis, can also be used [23,24,25]. Molecular-based methods tend to be more robust in detecting specific bacteria that may be present at low concentrations [23,25] but do not assess their viability, while enzymatic-based approaches are useful in estimating the overall metabolically active bacterial biomass [24]. Nevertheless, ATP analysis has several important limitations, namely, little precision in detecting low bacterial concentration, being unable to differentiate between intra- and extracellular ATP, and quantifications being biased by heterogeneous ATP concentrations per cell, which depend on bacterial species, cell size, and physiological states [24,26,27,28].

Single-cell analysis methods such as flow cytometry have been implemented for water quality assessment, specifically in wastewater treatment plants [26]. This is a fast, robust, and high-throughput method that may be used to quantify bacterial concentration in environmental matrices [29]. Its application coupled with the use of fluorophore dyes can enable differentiation between viable/metabolically active bacterial cells and dead/metabolically inactive cells [29].

Although chlorination is a known effective biocidal disinfection process against most microorganisms [30], there are few bactericidal/bacteriostatic activity data against fastidious bacteria able to enter dormancy. In this study, we monitored the metabolic inactivation of *M. bovis*, an example of a slow-growing bacterium able to enter dormancy, upon exposure to a commercially available chlorine-based water disinfectant used in veterinary settings by farmers and landowners. For biosafety reasons, we used *Mycobacterium bovis* BCG (biosafety level 2) as a proxy. In order to follow the decay of *M. bovis* BCG cells spiked in a mock environmental matrix, we applied a methodology we recently developed [17] that is based on single-cell technology—specifically, flow cytometry coupled with a resuscitation protocol—enabling the quantification of viable, dead, and dormant cells. The experimental design prompted the investigation of the inactivation effect exerted by a chemical disinfectant upon pathogenic mycobacteria’ viability under different disinfectant concentrations and environmental temperatures representing different environmental scenarios. The aims were to (i) appraise if this single-cell technology distinguishes different cell subpopulations (e.g., dormant) arising from exposure to disinfectants, thus enabling robust assessment of disinfection procedures’ effectiveness against metabolically heterogeneous (and so differentially susceptible) cells; and (ii) determine the most suitable disinfection conditions in different environmental backgrounds or seasons to enhance biosecurity in settings affected by *M. bovis* and multi-host TB.

## 2. Materials and Methods

### 2.1. Bacterial Growth and Culture Media

Biosafety level 2 *Mycobacterium bovis* BCG Tokyo (BCG) was used as a proxy of pathogenic *M. bovis*. Bacterial suspensions of BCG were cultured in Middlebrook 7H9 broth further supplemented with 0.5 g/L of sodium pyruvate (Sigma-Aldrich, St. Louis, MO, USA), 100 mL/L of 10× ADS, and 0.01% of Tween^®^80 (Sigma-Aldrich, USA), incubated at 37 °C (160 rpm) for four to five days. After, 50 mL of fresh liquid cultures were centrifuged at 3220× *g* for 10 min at room temperature, with subsequent pellet recovery. The cell pellet was washed three times with 1× PBS (pH 7.4), as previously described [17].

### 2.2. Preparation of Environmental Spiked Samples

Lake water and soil samples were collected from an urban area in Lisbon, where environmental contamination with pathogenic mycobacteria is not expected and was previously discarded via PCR. Samples were collected in hermetic containers and autoclaved three times. The sterilization efficiency was assessed by plate assays in Middlebrook 7H10 medium and by a semi-nested real-time PCR based on IS*6110*, as previously described [17].

A mock environmental matrix was prepared by mixing 20 g of soil sample and 40 mL of water sample. Spiking with 10^6^ BCG cells/mL (final concentration) followed, using BCG cell suspensions recovered during the exponential growth phase. Incubation with agitation under one of the three experimental temperatures (15 °C, 28 °C, or 37 °C) followed for 10 min to allow for a homogeneous distribution of metabolically active cells. After, a commercially available disinfectant (Aquasept^®^, Imporquímica, Portugal) was added to the mock environmental sample in one of three different concentrations: 5.25 mg/L, corresponding to half (0.5×) of the commercially recommended dosage; 10.50 mg/L, corresponding to the recommended (1×) dosage; and 21.00 mg/L, twice the recommended dosage (2×). Aquasept^®^ is composed of water-soluble sodium troclosene tablets (NaDCC). Each mock environmental sample exposed to a specific concentration of sodium troclosene was incubated without agitation under the three experimental temperatures for 168 h (7 days). Absence of agitation was meant as an emulation of natural environmental conditions in waterholes. Sterilized water was used instead of the disinfectant in the case of control environmental spiked samples. Non-spiked environmental samples were also processed as above as a control for extrinsic contamination. All conditions were tested in triplicate.

### 2.3. Cell Recovery from Mock Environmental Samples

In the following seven days, the mock environmental samples were subjected to analyses by the recovery of 1 mL of sample for metabolic cell evaluation. During the first hour, five samples were recovered at 0 h, 0.25 h, 0.50 h, 0.75 h, and 1 h. The instructions for Aquasept^®^ by the manufacturer recommend the use of the disinfectant after 0.50 h upon disinfection of water. After the regular evaluation during the first hour, the effect of prolonged exposure on BCG cell viability was monitored by subsampling each 12 h.

BCG cells were recovered from the mocked matrix using agitation for 5 min at 28 °C with the addition of 1 mL of cell recovery solution (1× PBS, 0.05% of Tween^®^80%, and 0.01% of sodium pyrophosphate). Following that, matrix suspensions were centrifuged at 3220× *g* for 30 min at room temperature and cell pellet was resuspended in 1.5 mL of 1× PBS, as previously described [17].

### 2.4. Cell Viability Staining assay and Flow Cytometry Analyses

The recovered cell pellet from the mock environmental samples was stained using 1.5 mL of cell suspension and 1 μL of 5-carboxyfluorescein diacetate acetoxymethyl ester (cFDA-AM, 2 mM, Thermo Fisher Scientific, Waltham, MA, USA) and incubated in the dark for 30 min at 37 °C, as previously described [17]. cFDA-AM is a permeant fluorophore, which, upon its entrance in viable cells, is cleaved by unspecific esterases, freeing carbofluorescein, that, when excited at 488 nm, emits green fluorescence.

Fluorescence was determined in CyFlow^®^ Space flow cytometer (Sysmex Partec, Goerlitz, Germany), which is equipped with a 20 mV solid-state laser emitting at 488 nm (blue fluorescence). Green fluorescence emitted by cFDA-AM was measured at 536/40 nm (FL1 channel). The flow cytometer was set to allow for an event rate of 1000–2000 events per second, thus allowing for single-cell analysis without the formation of cell aggregates. At acquirement, gains were set to a specific and adequate value kept for all analyses to ensure samples’ standardization. The trigger was set on Forward Scatter (FSC) with a logarithmic amplification. The data were registered using the CyFlow^®^ space operating software FloMax^®^ (Sysmex Partec, Germany) Version 2.2. The true volumetric absolute counting (TVAC) system present in the flow cytometer was used to perform cell quantification. Data analysis was performed using FlowJo™ Version 10 (Becton, Dickinson & Company, Franklin Lakes, NJ, USA) by comparing the spatial distribution of cell populations on a pseudocolor dot plot and the corresponding geometric mean of fluorescence intensity signal.

### 2.5. Evaluation of Dormant BCG Cells

At each time point in which viable cells were not detected, the presence of dormant cells was evaluated as previously described [17]. Resuscitation of dormant cells involved resuspending the mock environmental sample in Sauton medium (0.5 g/L of KH_2_PO_4_, 1.4 g/L of MgSO_4_·7 H_2_O, 4 g/L of L-asparagine, 0.5 g/L of sodium pyruvate, 0.05 g/L of ferric ammonium citrate, 2 g/L of sodium citrate, 0.1 mL/L of 1% ZnSO_4_·7 H_2_O [pH 7.0]), supplemented with 100 mL/L of 10× ADS and 0.01% of Tween^®^80, and incubation at 37 °C, at 200 rpm, for 5 days, as previously described [17]. Bacterial cell metabolism was evaluated by cFDA-AM staining as described above.

## 3. Results

Our study design involved testing three concentrations of a commercially available chlorine-based disinfectant (NaDCC, also called sodium troclosene) to understand (1) whether the recommended dosage by the manufacturer is efficient to completely kill all *M. bovis* BCG cells spiked in a mock environmental sample; (2) whether half the dosage of the disinfectant would be sufficient or, (3) on the contrary, if a higher dosage (e.g., twice) would be necessary. These experiments were performed at three different incubation temperatures as proxies of different environmental conditions, both geographical (locations with different mean temperatures) and seasonal (simulation of year-long temperature variation).

**Figure 1:**
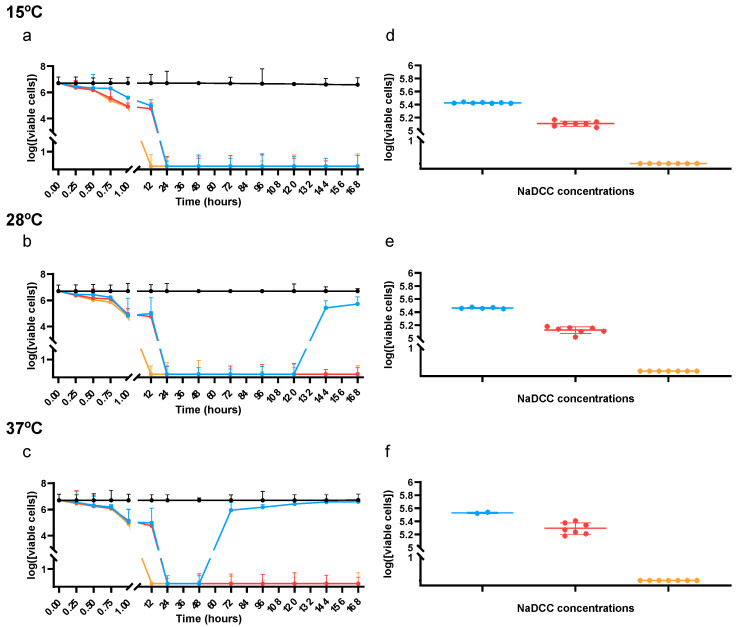
Disinfection efficiency of sodium troclosene (NaDCC) applied to mock environmental samples spiked with *Mycobacterium bovis* BCG Tokyo. The metabolic activity of environmentally recovered BCG cells was evaluated using 5-carboxyfluorescein diacetate acetoxymethyl ester (cFDA-AM). The logarithmic concentration of metabolically active cells throughout the 7-day experiment after exposure to three different concentrations of NaDCC is shown for cells incubated at (**a**) 15 °C, (**b**) 28 °C, and (**c**) 37 °C. Black: control condition without disinfectant; blue: 5.25 mg/L of NaDCC corresponding to half the recommended dosage; red: 10.5 mg/L NaDCC corresponding to the recommended dosage; yellow: 21 mg/L NaDCC corresponding to twice the recommended dosage. Vertical bars represent the standard deviation at each time point (n = 3). In each time point without detection of metabolically active cells (that is, times points in which the log concentration of metabolically active cells is zero in panels (**a**–**c**)), the presence of dormant cells was evaluated. The logarithmic concentration of metabolically active cells after the restoration of optimal growth condition for 5 days is shown for cells recovered from the (**d**) 15 °C experiment, (**e**) 28 °C experiment, and (**f**) 37 °C experiment. Blue: cells previously exposed to 5.25 mg/L of NaDCC; red: cells previously exposed to 10.5 mg/L NaDCC; yellow: cells previously exposed to 21 mg/L NaDCC. Results shown are the mean and standard deviation of three independent experiments.

At a lower temperature (15 °C), all concentrations of NaDCC (0.5×, 1×, and 2× recommended dosage) triggered a full loss of cell metabolic activity after 24 h upon exposure, without recovery of metabolic activity for the rest of the experiment (Figure 1a). At 28 °C, metabolic inactivation of BCG cells occurs after 24 h for all concentrations of NaDCC, with BCG cells resuming growth after 5 days upon exposure to the lower NaDCC concentration (0.5×) (Figure 1b). At a higher temperature (37 °C), which matches the optimal growth temperature of *M. bovis* BCG, metabolic cell inactivation was registered after 24 h for all concentrations of NaDCC, but BCG cells resumed growth after two days upon exposure to a lower concentration (0.5×) of NaDCC (Figure 1c).

The presence of dormant cells was evaluated at all time points in which the metabolic activity of BCG cells could not be detected. At all tested temperatures, BCG cells were able to regain their metabolic activity to values close to the control (no disinfectant) group’ (10-fold difference) when the lowest (5.25 mg/L, 0.5×) and the commercially recommended dosage (10.50 mg/L, 1×) of NaDCC were used (Figure 1d–f). However, the application of a higher dosage (twice the commercially recommended dose; 21.00 mg/L) of NaDCC was able to efficiently disinfect BCG cells, hampering the regain of metabolic activity upon resuscitation-promoting conditions (Figure 1d–f). The concentration of metabolically active BCG cells after resuscitation assays was independent of the exposure time to NaDCC since all tested time points showed similar results (Figure 1d–f).

Thus, we found that temperature does not influence the efficiency of NaDCC if the higher, double dosage is used (21.00 mg/L), because of the total disinfection of BCG cells 12 h after exposure, and after 7 days, with BCG cells being unable to regain metabolic activity even after optimal growth conditions are restored.

## 4. Discussion

The control of pathogens able to survive in the environment independently from their host remains a significant challenge for effective disease management in both resilient and vulnerable scenarios; thus, disinfection, biocontainment, and monitoring tools are utterly needed for farmers and managers to reduce the impacts of pathogens with environmental cycles.

Livestock health is influenced by drinking water quality, with studies showing contradictory results regarding cattle avoidance of water supply with fecal contamination. Some studies report the avoidance of such contaminated water [31], while others have shown high tolerance to high bacterial loads in drinking water [32]. Nevertheless, the consumption of drinking water previously contaminated with faces is known to reduce animal productivity due to a general decline in health status and the development of waterborne and foodborne diseases [31,33,34].

To tackle microbial contaminants, several methodologies have been implemented, with chlorination being one of the most common water disinfection treatments available [35]. This process consists of the addition of chlorine or chlorine compounds to water. When added to water, both chlorine and hypochlorous acid are neutral molecules that can penetrate bacteria’s surface, leading to the disintegration of the cell membrane and cell wall due to lipid breakdown and collapse [35]. After, these chlorine compounds also denature intracellular proteins [35]. This leads to a biostatic or biocidal effect on bacteria, depending on chlorine concentration, bacterial genus, bacterial morphotype, and cell wall structure and composition.

The effect and efficiency of such disinfectant procedures need to be evaluated to ensure water security. Several methodologies are usually applied in the evaluation of microbial contamination in water, with flow cytometry showing to be a promising single-cell technique for environmental bacterial community analyses [26,29].

In this study, we examined, via an innovative single-cell workflow, the effectiveness of managing an environmentally contaminated hydric scenario with a disinfectant. We used a chemical disinfectant, NaDCC, and a combined laboratory trial to experimentally reduce the bio-burden of a slow-growing bacterium, the etiological agent of animal TB, *M. bovis*, in the environment. This workflow, based on flow cytometry and cFDA-AM staining, was able to detect heterogeneity in the metabolic activity of BCG cells during seven days upon exposure to NaDCC. This assessment, based on the monitoring of metabolic activity, has the advantage of a single-cell method, with fast, robust, and high-throughput outputs compared with traditional methodologies, such as MPN, multiple tube fermentation, plate count, and membrane filtration [26,29]. Moreover, when compared to molecular-based methods, such as DNA-based or enzymatic-based ones, this workflow enables the detection of single bacterial cells, instead of bacterial DNA or enzymes, which are unreliable proxies to estimate cell burden due to the presence of environmental loose DNA and extracellular enzymes [23,24,25]. Furthermore, our workflow relies on a parallel resuscitation step that (1) enables the quantification of bacterial cells entering a dormancy state upon the deleterious effects of NaDCC or other disinfectants and (2) differentiates those cells from the ones that are unable to recover metabolic activity, contrary to all other previously reported methods, including other previously reported flow cytometry-based methodologies, which do not differentiate between dormant and dead cells [26].

A previous study focusing on the application and efficiency of disinfectant products to cattle drinking water also tested NaDCC (2 mg/L) [35]. It was found that NaDCC did not have a complete lethal effect, in which surface water had a reduction from 10^7^ to 10^2^ CFU/mL of total viable cells based on a plate count method [35]. However, in that work, NaDCC was tested at a much lower concentration compared with the one used in the present study [35]. Other authors tested different chlorine-based disinfectants, such as NaClO (15% *m*/*v*) [32]. Chlorination and acidification (with H_3_PO_4_) increased free residual chlorine in water, and chlorination reduced total coliform counts in cattle drinking water [32]. Furthermore, the disinfection treatment applied to fattening dairy beef bulls’ drinking water seemed to improve growth performance without side effects on health or nutrient digestibility [32].

In contrast with these studies, the effects of chorine-based disinfection upon mycobacteria in veterinary settings or upon fastidious bacteria growing in natural substrates, such as water and sediments from dams/waterholes, had not been reported. The atypical cell wall of mycobacteria species, highly rich in long-chain lipids, confers resistance to a large variety of disinfectants [36,37]. When added to water, chlorine, as a neutral molecule, can penetrate the bacterial surface, leading to the partial disintegration of the plasma membrane and cell wall due to lipid breakdown [35]. After, it causes denaturation of intracellular proteins, leading to biostatic or biocidal effects, depending on their concentration and the bacterial cell structure [35]. In our work, results suggest that lower dosages of NaDCC (<10.50 mg/L) exert reversible damage upon BCG cell structure and function, while twice the recommended dose (21.00 mg/L) hampers essential cell metabolism and cell division, leading to irreversible lethal effects.

The resuscitation conditions used in the present study mimic the optimal growth conditions of *M. bovis*, including incubation at 37 °C, oxygenation, and a rich broth medium. In natural infecting conditions, and upon host entry, *M. bovis* is exposed to rather constant temperatures, but also to oxygen and nutrient depletion after granuloma formation [38], leading to slower metabolism and dormancy. Subsequent metabolic reactivation of mycobacteria upon host immunosuppression is described [38]. In the natural environment, the range of atmospheric temperatures is usually wide, and the oxygenation of waterholes is limited. So, although we registered the reactivation of cell division in BCG cells after NaDCC exposure, this might not be verified in suboptimal field conditions. Further, we tested the inactivation of pure cultures of BCG in a mock environmental matrix. The application of NaDCC in the field, where biodiverse microbial communities thrive, may show different biocidal effects against mycobacteria along with other microbial co-contaminants.

Chlorine-based agents have been proven to maintain both the organoleptic characteristics of drinking water and healthy animal metabolism. Nevertheless, increased concentrations of NaDDC may lead to other adverse side effects besides killing mycobacteria, causing, for instance, loss of biodiversity in waterholes, with effects on invertebrate communities and implications for the natural ecosystem. Chlorine-based disinfectants have been previously described to have a long-term impact on ecosystem health, posing a high ecotoxicological risk for fish, algae, and daphnia taxonomical groups in surface, river, and lake water environments [39]. Further assessment is thus needed.

This study further highlights the lack of complete effectiveness of disinfection measures, despite the apparent inactivation of microorganisms. This residual community, maintaining the capacity to grow after disinfection, deserves special attention, particularly from a microbial community structure perspective [40], in which single-cell methodologies, such as flow cytometry, can possess great applicability [29].

Overall, this study highlights the high resolution of flow cytometry coupled with resuscitation assays to accurately monitor the efficacy of biocontainment strategies, particularly in the case of metabolically heterogeneous and dormant bacteria, thus supporting data-driven environmental management of contaminated settings.

## 5. Conclusions

In the Iberian Peninsula, land management from the perspective of livestock farming and big game hunting ensures the economic viability of many estates. This implies a varying degree of artificialization and supplementation to keep a high abundance of species near the property. Landowners in epidemiological risk areas for animal TB, where disease prevalence is high, are told to apply water disinfectants in waterholes and in animal drinking water supplies to empirically prevent water contamination. However, the efficacy of these procedures in preventing TB transmission remains to be elucidated. With this work, we show the benefit of using single-cell approaches to monitor these fastidious bacteria, not only to assess the experimental biocidal effects of disinfectants in vitro but also to assess microbial contamination in field conditions, informing the implementation of biocontrol measures. While we demonstrate that, at the recommended dosage, commercial NaDCC is unable to fully inactivate *M. bovis* BCG cells from a mock environmental matrix mimicking natural and artificial waterholes in veterinary/agricultural settings, we show that increasing to twice the chorine biocide concentration is enough to prevent mycobacteria cell function and regrowth. The employment of this easy-to-implement disinfection measure, together with other actions already being implemented in the field in the context of animal TB control, might prevent the dissemination of pathogens with environmental cycles in vulnerable semi-arid systems and, hence, new infections.

## Data Availability

Data are contained within the article.
